# Impact of COVID-19 on Neuropsychiatric Disorders

**DOI:** 10.3390/jcm11175213

**Published:** 2022-09-03

**Authors:** Niloufar Zia, Parsa Ravanfar, Sepideh Allahdadian, Mehdi Ghasemi

**Affiliations:** 1 Department of Psychology, Lesley University, Cambridge, MA 02138, USA; 2 Department of Psychiatry, University of Texas Southwestern, Dallas, TX 75390, USA; 3 Department of Neurology, Penn State Milton S. Hershey Medical Center, Hershey, PA 17033, USA; 4 Department of Neurology, University of Massachusetts Chan Medical School, Worcester, MA 01655, USA

**Keywords:** coronavirus disease 2019 (COVID-19), severe acute respiratory syndrome coronavirus 2 (SARS-CoV-2), neuropsychiatry, neurodegenerative diseases, neurodevelopmental diseases

## Abstract

Since the Coronavirus disease 2019 (COVID-19) pandemic, caused by severe acute respiratory syndrome coronavirus 2 (SARS-CoV-2), many studies have shown that besides common COVID-19 symptoms, patients may develop various neuropsychiatric conditions including anxiety, mood disorders, psychosis, neurodegenerative diseases (e.g., dementia), insomnia, and even substance abuse disorders. COVID-19 can also worsen the patients underlying neuropsychiatric and neurodevelopmental conditions during or after the system phase of disease. In this review, we discuss the impact of SARS-CoV-2 infection on development or status of neuropsychiatric conditions during or following COVID-19.

## 1. Introduction

**Figure 1:**
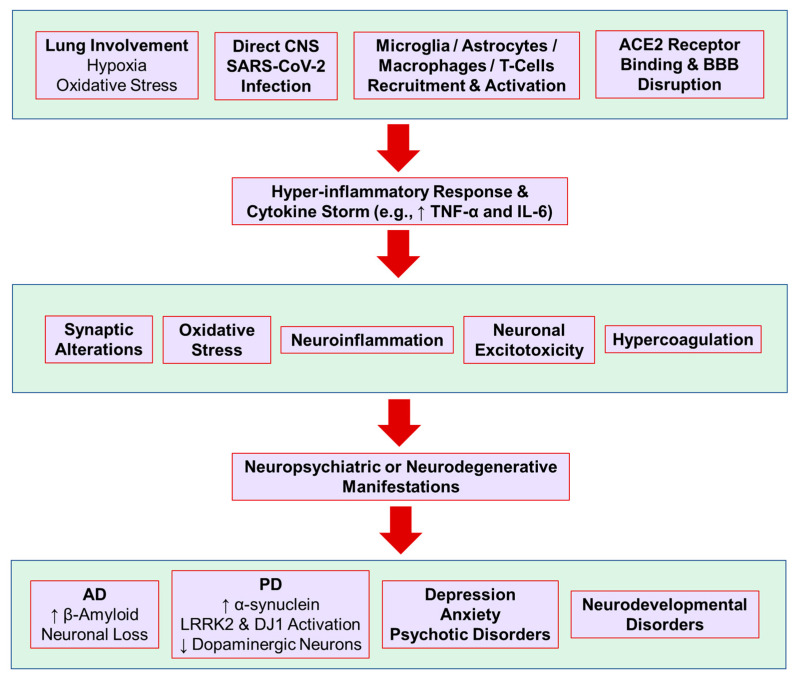
Neuropsychiatric manifestations and possible underlying mechanisms after SARS-CoV-2 infection. ACE2, angiotensin-converting enzyme 2; AD, Alzheimer’s disease; BBB, blood-brain barrier; IL-6, interleukin-6; LRRK2, leucine-rich repeat kinase 2; PD, Parkinson’s disease; TNF-α, tumor necrosis factor-α.

In December 2019, a novel Coronavirus named Severe Acute Respiratory Syndrome Coronavirus 2 (SARS-CoV-2) was identified in Wuhan, China. Soon after it became an epidemic throughout the world. SARS-CoV-2 has some spikes on its surface which are the membrane-anchored tiners consisting of receptor binding s1 and membrane-fusion s2 segment. The Cov name originated from these spikes. S1 segment consists of the receptor binding domain (RBD) that causes pathogenicity and infects the host cell via binding to the angiotensin-converting receptor-2 (ACE-2) on all tissues [1,2]. RBD has greater affinity for ACE-2 on cells of the ileum, kidney, heart, brain, lung, and vasculatures. It is responsible for the different manifestations it has, including respiratory disease and mild pneumonia; its usual symptoms include fever, shortness of breath, cough, and fatigue [3]. Besides systemic manifestation, accumulating reports indicate that patients with COVID-19 may develop a variety of neuropsychiatric conditions during or after COVID-19 (Figure 1) [4,5,6,7]. SARS-CoV-2 infection may also affect the patients’ current neuropsychiatric conditions in various ways. Given the high burden of neuropsychiatric conditions on society besides COVID-19 itself, in this review, we will discuss the impact of SARS-CoV-2 infection on new-onset and current neuropsychiatric conditions in patients with COVID-19.

## 2. Neuropsychiatric Complications of COVID-19

Acute neuropsychiatric presentations and potentially long-term complications have been reported in people infected with COVID-19 as well as those who recovered from it [5]. In a large study using the TriNetX Analytics Network (a federated network recording anonymized data from electronic health records in 62 healthcare organizations, primarily in the United States [US]) and including 62,354 patients with confirmed diagnosis of COVID-19 [8], it was shown that COVID-19 was associated with increased incidence of a first psychiatric diagnosis in the following 14 to 90 days compared with six other health events (i.e., influenza, other respiratory tract infections, skin infection, cholelithiasis, urolithiasis, and a large bone fracture). The hazard ratios were greatest for anxiety disorders, insomnia, and dementia. The incidence of any psychiatric diagnosis in the 14 to 90 days after COVID-19 diagnosis was 18.1%, with 5.8% having a first diagnosis (e.g., first diagnosis of dementia 1.6% in patients older than 65 years) [8,9]. There is a wide range of underlying etiologic factors both within and beyond the CNS that lead to neuropsychiatric sequelae (Figure 1) [7].

In two large sample retrospective studies of 40,469 patients who recovered from COVID-19 in the TriNetX database, diagnosis of anxiety (and related disorders) and mood disorders was established in 4.6% and 3.8%, respectively, on or within one month after diagnosis of COVID-19 [6] (Table 1). Another retrospective study on 44,779 COVID-19 patients without previous psychiatric illness in the TriNetX Analytics Network revealed that the rate of all diagnoses of psychiatric disorders (i.e., including relapses) was higher within 14 to 90 days after COVID-19 diagnosis than after control health events (i.e., influenza, other respiratory tract infections, skin infection, cholelithiasis, urolithiasis, and a large bone fracture) [9]. The most common psychiatric diagnosis after COVID-19 diagnosis was anxiety disorder (12.8%, 95% confidence interval [CI] 12.4–13.3), followed by mood disorders (9.9%, 9.5–10.3). The probability of a first diagnosis of mood disorder within 14 to 90 days after COVID-19 diagnosis was 2% (95% CI 1.7–2.4), with depressive episode as the most common first diagnosis of mood disorder (1.7%, 95% CI 1.4–2.1) [9]. In another retrospective cohort study on 236,379 survivors of COVID-19 in the TriNetX Analytics Network, estimated incidences for first-time anxiety disorders in the following 6 months in the whole cohort and those admitted to intensive care unit (ICU) were 17.39% (95% CI 17.04–17.74) and 19.15% (95% CI 17.90–20.48), respectively [8]. Moreover, the estimated incidences for first-time mood disorders in the following 6 months in this study were 4.22% (95% CI 3.99–4.47) and 5.82% (95% CI 4.86–6.97) [8]. The prevalence of self-reported symptoms of depression and anxiety was much higher. Studies using self-report tools have suggested a markedly greater frequency of depressed mood (29.2%), anxiety, and post-traumatic anxiety symptoms (20.8–96.2%) in different countries including China and Italy [10,11,12,13] (Table 1).

There are multiple factors that contribute to the development of mood and anxiety disorders associated with COVID-19. These factors can be categorized into three domains: contextual factors and life stressors, disease-related reduction in quality of life such as fatigue and breathlessness, and biological factors impacting the brain such as neuro-immunological phenomena. Based on the evidence provided by Taquet et al., this higher occurrence of first-time mood and anxiety disorders cannot be solely attributed to the contextual factors such as economic and social adversities/challenges/stressors associated with COVID-19. Here, we focus on the disease-related pathophysiological mechanisms that may account for the occurrence of these psychiatric manifestations. The association between COVID-19 and depression and anxiety can be due to neurotropism of the virus, or the immunological reactions of the body such as cytokine storm [18]. In a study by Mazza et al. in Italy, there was a significant association between systemic immune-inflammation index (SII) with measures of anxiety and depression [12]. COVID-19 has been characterized by an exaggerated inflammatory response known as a “cytokine storm”, and inflammatory cytokines have been associated with depression [19]. Conversely, the presence of a previous diagnosis of mood disorder is associated with higher mortality rates after prolonged hospitalization [20].

First-time psychosis may also occur among patients with COVID-19 as assessed in the TriNetX Analytics Network as well as systematic reviews of case reports and case series from different regions worldwide [8,9,21]. Although there has been shown a low probability of being newly diagnosed with a psychotic disorder in the 14 to 90 days after COVID-19 diagnosis (0.1%, 95% CI 0.08–0.2), broadly similar to the probability after control health events [9], the estimated incidences of psychotic disorders within 6 months after COVID-19 was higher by 1.40% (95% CI 1.30–1.51). It was even more increased in those patients initially admitted to the ICU due to COVID-19 (2.77%, 95% CI 2.31–3.33) [8]. A more recent systematic review of case reports and case series of 57 patients with COVID-19 (collected from six electronic databases including PubMed, Scopus, Web of Science, PsycInfo, PsycArticles, and CINAHL) also reported that the mean age for onset of psychotic symptoms (predominantly delusions and hallucinations) was the early 40s (men: 43.4 and women: 40.3), more than two thirds (~69%) of patients did not have any prior psychiatric disorders [21]. Only 26.3% of patients presented with moderate-severe COVID-19-related disease and complications. Overall, psychotic symptoms resolved markedly in 63.2% of cases after treatment with antipsychotics, benzodiazepines, valproic acid, and electroconvulsive treatment [21]. COVID-19-related new-onset psychosis and mania have been also reported in children and adolescents, even in those with asymptomatic COVID-19 infections as described in some case reports in the U.S. [22].

New onset dementia and cognitive dysfunction can occur in relation with COVID-19 [8,9,16,23]. A retrospective analysis of 50 patients with COVID-19 in the U.S. found cognitive impairment in 26% of cases [23]. Another study on 153 COVID-19 cases in the United Kingdom (UK) also showed that overall, 23 (15.3%) patients developed neuropsychiatric disorders related to COVID-19, among which 10 (43%) patients had new-onset psychosis, 6 (26%) had a neurocognitive (dementia-like) syndrome, and 4 (17%) had an affective disorder [16]. Further larger cohort studies showed that the estimated incidence of dementia during the first 14 to 90 days after a diagnosis of COVID-19 is 0.44% (95% CI 0.33–0.60); which was higher in those patients older than 65 years (1.6%, 95% CI 1.2–2.1), with a hazard ratio (HR) between 1.89 and 3.18 [9]. The estimated incidence of new dementia in the following 6 months was even higher by 0.67% (95% CI 0.59–0.75) and again with an increased rate in those patients initially admitted to ICU due to COVID-19 (1.74%, 95% CI 1.31–2.30) or in those who had encephalopathy by 4.72% (3.80–5.85) [8].

Both short-term (i.e., within 14 to 90 days) and 6-month onset of other neuropsychiatric conditions, such as insomnia and substance use disorder, may rise in the COVID-19 population as assessed in the TriNetX Analytics Network [8,9]. A post-discharge evaluation of 120 COVID-19 patients in France after an average of 110.9 days following admission showed that the most frequently reported persistent symptoms were fatigue (55%), dyspnea (42%), loss of memory (34%), difficulty in concentration (28%), and sleep disorders (30.8%) [24]. The probability of a first diagnosis of insomnia in the 14 to 90 days after COVID-19 diagnosis was further shown to be 1.9% (95% CI 1.6–2.2), more common than after controlled health events (HRs 1.85–3.29), in agreement with predictions that circadian disturbances will follow COVID-19 infection [9,25]. Close to 60% of the insomnia diagnoses were not accompanied by a concurrent diagnosis of an anxiety disorder [9]. The estimated incidence of first insomnia within 6 months post-COVID-19 was 2.53% (95% CI 2.37–2.71) [8]. The estimated incidence of new substance use disorder within 6 months following COVID-19 was 1.92% (95% CI 1.77–2.07) [8]. Overall, the above data indicate that COVID-19 is followed by remarkable rates of long-term neuropsychiatric diagnoses.

## 3. Impact of COVID-19 on Neuropsychiatric Disorders

The COVID-19 virus infection and the impact of the virus on society causes several different sequels and comorbidities in physical and mental health [26]. Infection with COVID-19 may cause the worsening of neuropsychiatric disorders and/or the development of neuropsychiatric disorders [26,27]. In addition, the societal consequences of the pandemic (i.e., isolation and quarantine) have caused the worsening and development of neuropsychiatric disorders [26]. Examples of relevant neuropsychiatric disorders in the context of COVID-19 include depression, anxiety, delirium, mood compulsivity, cognitive impairment, and obsessive-compulsive symptoms [26,28]. There are a variety of mechanisms that likely cause the worsening of the development of neuropsychiatric disorders in the context of COVID-19 infection and the ongoing pandemic [26].

Recent research examining the impact of COVID-19 infection on mental health functioning suggests that the virus causes an inflammation of the CNS which, in turn, may stress mental health processes and functioning through cytokine secretion [26]. Other research suggests that COVID-19 increases the risk of or exacerbates neuropsychiatric disorders via hypoxemia, which is common among individuals infected with COVID-19 [29,30]. Moreover, other research identifies additional ways by which COVID-19 infection and the pandemic negatively impact mental health (e.g., isolation, changes in social support, economic stressors) [26].

Individuals with pre-existing neuropsychiatric disorders and symptoms are likely at greater risk of poor mental health outcomes in the context of COVID-19 [26,31]. Indeed, the worldwide virus appears to affect people differently based on their baseline psychiatric functioning. As such, individuals with mood disorders, anxiety disorders, and psychotic disorders, may have a different baseline of psychiatric functioning and could be at higher risk of developing neuropsychiatric disorders or experiencing a worsening of neuropsychiatric symptoms following COVID-19 infection, as assessed in different studies and regions including the U.S. and Brazil [8,9,26,31,32].

Individuals with neuropsychiatric disorders demonstrate different and worse symptoms and functioning as compared to their counterparts in the context of the ongoing COVID-19 pandemic [26,33]. Specifically, individuals with pre-existing mood and anxiety disorders report increased stress and fear of pollution in the current pandemic [31]. In addition, these symptoms reached clinically significant or concerning levels. Among individuals with a pre-existing neuropsychiatric disorder who contracted COVID-19, many experienced an exacerbation of mental health symptoms [26,31,34,35]. Specifically, individuals may have experienced serious neuropsychiatric complications, including delirium, cognitive impairment, significant mood alterations, or even psychosis [26,31,32]. Indeed, even among people without pre-existing neuropsychiatric disorders, delirium occurs in most individuals who contract COVID-19 [26,36].

### 3.1. Anxiety Disorders

Individuals with anxiety disorders are at risk of poorer outcomes in the context of the COVID-19 pandemic [34]. Using an online subject pool from the U.S. and Canada, researchers found that individuals with anxiety-related disorders experienced higher stress as well as greater fear, socioeconomic consequences, xenophobia, and traumatic stress as compared to individuals with mood disorders and individuals without clinically significant mental health concerns in the context of the COVID-19 pandemic [34]. As such, individuals with anxiety disorders are at greater risk of certain poor outcomes even as compared to individuals with mood disorders in the context of the ongoing COVID-19 pandemic [34]. Regarding mechanisms behind the relationship between anxiety disorders and poor outcomes, individuals with anxiety disorders were more likely to engage in effortful isolation and ineffective coping strategies [34]. Perhaps these behaviors, in combination with other associated behaviors, increased the risk of certain poor outcomes for individuals with anxiety disorders as opposed to their counterparts [34]. The increased risk of poor outcomes across several domains (i.e., psychological or economic) among individuals with anxiety disorders in the context of the COVID-19 pandemic cannot be ignored. There is a need for research, intervention, and policy to address the increased risk and poor outcomes among this population.

### 3.2. Mood Disorders

Based on a recent meta-analysis, individuals with a pre-existing mood disorder have a significantly higher chance of hospitalization and death as compared to those without a pre-existing mood disorder following COVID-19 infection [37]. These meta-analytic results highlight the vulnerability of individuals with pre-existing mood disorders for poor health outcomes in the context of contracting COVID-19. As such, researchers and policymakers should consider those with pre-existing mood disorders when creating policy regarding vaccinations and other relevant public health decisions [37]. Mood disorders may confer a greater risk of susceptibility to COVID-19 for a variety of reasons. Individuals with pre-existing mood disorders experience increased psychological stress during the COVID-19 pandemic, which was further associated with maladaptive life and behavioral changes [34,38]. Certain mood disorders are associated with a greater risk of poor outcomes. For instance, a study in Australia demonstrated that specifically men with bipolar disorder are the most at-risk group among individuals with mood disorders regarding risk of depression and financial concerns [38]. As such, it is important to consider how individuals with pre-existing mood disorders may be at risk of certain poor health and psychological outcomes in the context of the ongoing COVID-19 pandemic.

### 3.3. Neurodevelopmental Disorders

Neurodevelopmental disorders typically emerge during early-to-middle childhood and may result in functional impairment and/or limitations regarding neuropsychological, cognitive, and adaptive development. Examples of neurodevelopmental disorders include autism spectrum disorder (ASD), attention-deficit/hyperactivity disorder (ADHD), intellectual disabilities, and specific learning disabilities [39].

Unfortunately, children with neurodevelopmental disorders (e.g., ASD and ADHD) are at higher risk of poor mental health and difficulties with functioning than their counterparts in the context of the COVID-19 pandemic, as evidenced by a cross-sectional parent-reported study in the UK [40]. Indeed, children with neurodevelopmental disorders experienced an increase in emotional difficulties and conduct problems as well as a decrease in prosocial behaviors during the pandemic [40]. Depression and anxiety among children and their parents with ASD have increased as reported by studies in different countries such as the US and Switzerland [41,42]. Among children with neurodevelopmental disorders studied in the UK, female children with ASD experienced the highest emotional symptoms [40]. The severity of neurodevelopmental symptoms increased during the course of the pandemic among children and adolescents in the U.S. [43]. In addition, a recent study on 238 adolescents (ages 15–17 years) from two sites in the Southeastern and Midwestern U.S. showed that associated behavioral considerations, including opposition/defiance and impulsivity, also increased during the pandemic [44]. The stressors and increased symptomatology of children with neurodevelopmental disorders also impacted parents; indeed, parents of children with neurodevelopmental disorders reported worse mental health than their counterparts during the pandemic in the UK [40].

Research has provided potential mechanisms or avenues by which individuals with neurodevelopmental disorders experienced worse outcomes in the context of the pandemic. Specifically, individuals with neurodevelopmental disorders experienced closures in or abrupt disruption of their health services [45], which likely caused decreases in physical and mental health [46,47]. The lack of access to physical exercise during lockdown and quarantine(s) may have been particularly detrimental to individuals with neurodevelopmental disorders as exercise may be used to regulate several symptoms of neurodevelopmental disorders [46]. Researchers have also postulated that boredom and associated decreases in motivation have engendered rises in depression and poor outcomes among children and young people with neurodevelopmental disorders [48]. In addition, changes in remote and hybrid schools were associated with increased dropout rates from school among children and young adults with ADHD in the U.S. [43].

Certain aspects of the ongoing pandemic may have increased risk of poor outcomes among children with ASD specifically. Indeed, individuals with ASD may have certain vulnerabilities that were exacerbated by the pandemic [48]. Quarantine and isolation policies in response to the pandemic caused severe disruption to the routines and services typically accessed by people with ASD [49]. In some cases, individuals with ASD lost complete access to services partially because individuals with ASD were not considered a marginalized population [20]. In addition, the evaluation of individuals with suspected ASD was also interrupted and services and diagnoses were delayed [20]. As such, individuals with ASD and individuals yet to be diagnosed with ASD experienced specific stressors that likely impeded effective intervention and support services. As a result, the research described above regarding poor outcomes among individuals with ASD and their families is not surprising.

Although initial research indicates that the negative impact of quarantine and lockdown on mental and physical health does not appear to be long-lasting among children and young people with neurodevelopmental disorders, longitudinal research has not yet been conducted to examine the long-lasting impact of COVID-19 on individuals with neurodevelopmental disorders [44].

### 3.4. Psychotic Disorders

Psychotic disorders are characterized by cognitive and perceptual dysfunction, usually hallucinations or delusions [50]. Psychotic disorders may be accompanied by mood disturbances and can be caused by [50]. In the U.S., between 0.25% and 0.64% of the population is diagnosed with a psychotic disorder [51]. However, over the course of the pandemic, the incidence of psychotic disorders and symptoms has risen [51]. Moreover, the risk of psychosis among individuals with COVID-19 is higher than the average population, with between 0.9% and 4% of individuals with COVID-19 experiencing psychosis [51].

As among individuals with neurodevelopmental disorders, services and supports for individuals with psychotic disorders were severely disrupted over the course of the pandemic. In addition, because of the symptoms and vulnerabilities associated with psychotic disorders, this population is highly vulnerable to the changes in routine and access to care caused by the COVID-19 pandemic [51]. Individuals with psychotic disorders may have experienced disruptions in their access to medication and face-to-face services [51,52,53,54]. These changes and barriers to services caused significant decompensation among many individuals with psychotic disorders and were also associated with increased paranoia and anxiety [51,52]. Decompensation refers to a significant deterioration in psychological functioning and an increase in severity of symptoms that is not typical [51]. Because of the transition to telehealth and phone services, providers may not have been able to accurately track and identify patients’ decompensation over the course of the pandemic [51]. As such, individuals with psychotic disorders may have been uniquely at risk for experiencing worsening symptoms without proper support or intervention [51].

A single-center retrospective and observational study in Spain found that SARS-CoV-2 infection has also increased risk of and severity of psychosis symptoms [55]. Indeed, psychosis (e.g., delusions) has emerged among individuals with no history of these symptoms following infection with the virus [55]. However, it is unclear to what extent psychosis following infection with COVID-19 is caused by the virus itself versus the medications used by health providers to treat the virus [55]. Certain researchers posit that COVID-19 causes inflammation in the central nervous system, which then causes individuals to experience psychosis [55]. Regardless of the exact cause, individuals with COVID-19 are at increased risk of experiencing psychosis, even those without a prior history [55]. More research studies are needed to understand the cause of psychosis among individuals with COVID-19 as well as assessment and intervention strategies.

### 3.5. Cognitive Disorders

Cognitive disorders are overall characterized by executive function impairment and are associated with difficulties with organization, regulation, and perception [56]. Individuals with cognitive disorders may experience difficulties with processing speed, reasoning, decision-making, awareness, attention, learning, impulsivity, memory, or language [56]. More than 16 million individuals in the United States are currently diagnosed with a cognitive disorder [57].

Individuals with cognitive disorders are at significantly higher risk of contracting the virus given baseline difficulties with executive functioning [54]. Indeed, because individuals with cognitive disorders may struggle to care for themselves, it may be harder for them to adhere to safety standards regarding isolation, social distancing, and quarantine [58]. As part of this concern, individuals with cognitive disorders may live in collective housing or treatment units and the functioning of these structures has been severely impacted by the pandemic. As such, it is highly likely the functioning of individuals within these communities has also been severely affected. In addition, such facilities for individuals with cognitive disorders may allow the virus to spread easily across patients and staff putting individuals with cognitive disorders at even higher risk of contracting the virus [59]. If infected with COVID-19, individuals with cognitive disorders may also have worse outcomes, especially if their access to caregivers or supervision is limited in the context of ongoing social distancing and quarantine policy [58,60]. Research is needed to identify and develop standards for communal living facilities for individuals with cognitive disorders that prioritizes the delivery of services and the safety of patients.

Intellectual disabilities are a subset of cognitive disorders. Unfortunately, the health and well-being of individuals with cognitive disorders has been negatively impacted by the ongoing pandemic. Specifically, research indicates that mental health and physical activity have significantly decreased among children and young adults with both physical and intellectual disabilities [47]. Decreases in physical activity were due to quarantine and isolation policies as well as the closing of community centers and exercise facilities [47]. Among parents of children with intellectual disabilities, 90% reported worsening mood and increased behavioral problems among their children [47]. Although this study identified the potential for the development of solutions to the problems and barriers experienced by individuals with intellectual disabilities, no such solutions have been systematically implemented [47]. Thus, both research and advocacy are needed to address the problems and outcomes among individuals with intellectual disabilities over the course of the ongoing pandemic.

### 3.6. Neurodegenerative Disorders

Studies have revealed that ACE-2 is also expressed on neurons, glial cells, epithelial cells of blood-brain barrier (BBB), as well as oligodendrocytes. Interestingly a high concentration of substantia nigra may help the virus to enter, which itself may be the cause of reported neuropsychiatric sequelae of the infection [61]. Varieties of neurological symptoms including headache, ageusia, anosmia, and different forms of neurodegenerative disorders have been reported as consequences of SARS-CoV-2 infection [62]. Inflammation as a consequence of viral entry to the brain can cause oxidative damage and apoptosis of the cells in the brain, which has been reported as a cause of neurodegeneration and neurodegenerative disease [63]. Alzheimer’s disease (AD) and Parkinson’s disease (PD) are two neurodegenerative disorders reported to be caused by prolonged inflammation in patients infected by SARS-CoV-2 as a possible postinfectious manifestation. Inflammatory processes are also involved in other neurodegenerative disorders such as progressive supranuclear palsy syndrome (PSPS) [64], corticobasal syndrome (CBS) [64], and multiple system atrophy-parkinsonian type (MSA-P) [65]. COVID-19 has been reported to affect the international classification of functioning, disability, and health functioning in patients with MSA [66]. Accumulating evidence also suggests that SARS-CoV-2 infection can induce neurodegenerative disorders [67].

In view of the above, SARS-CoV-2-induced reactive oxygen species (ROS) caused by oxidative damage has been reported to cause accumulation of the amyloid beta (Aβ) proteins which itself is involved in the pathogenesis of AD. In addition, increased tau level as a result of neuroinflammation followed by viral entry to the brain has been reported as another possible cause of cognitive impairment within patients of SARS-CoV-2 [67,68]. COVID-19 incidence and complications are increased in patients with AD and related dementia. Patients with AD or related dementias have a cognitive impairment which causes difficulty understanding and remembering the recommendations. This disease is also more associated with other comorbidities such as cardiovascular disease, which may be another reason for high mortality of COVID-19 in these patients (90%) [69,70]. It has been also reported that APOE4 isoform of AD which causes a decreased amount of APO has increased the risk for COVID-19 infection and progression [71].

Several possible pathogeneses have been reported as a cause of PD in COVID-19 patients. Vulnerability of the basal ganglia and dopamine-rich region as well as the neuroinflammation caused by SARS-CoV-2 has been reported as a possible cause of failure of dopamine synthesis in COVID-19 patients [67]. Moreover, DJ1 and Leucine-rich repeat kinase 2 (LRRK2), key proteins in dopamine regulation and oxidative reaction, can be affected by SARS-CoV-2, resulting in dopamine dysregulation and inflammation in substantia nigra and α-synuclein aggregation [67,72].

Apart from the effect of COVID-19 on patients’ access to different medical and psychological care, decreased physical activity and family support [73] and different neuropsychiatric outcomes have been reported that may be due to dopamine depletion in PD patients as a neurotransmitter to help with adoption to different changes in healthy individuals, which is lacking in PD patients. Anxiety and stress may remain and become chronic due to lack of adaptation as a result of dopamine depletion [74]. There are three different hypotheses about the effect of COVID-19-induced stress in PD patients. Stress can interfere with the levodopa effect and reduce it [75]. In addition, chronic stress has been revealed to decrease the dopamine activity within the rodent’s ventral tegmentum compared to healthy controls and has been reported as a cause of neurodegeneration in PD and AD [76]. Emotional stress as a trigger for freezing gait has been reported as well [77]. One important aspect of COVID-19 infection is dehydration, diarrhea, fever and decrease in water intake which may be another important aspect to consider when adjusting the medication dosage. However, COVID-19 infection has been reported as a trigger for worsening of PD motor and nonmotor symptoms. Although, the patients with higher age and duration of illness had been reported to have more mortality risk compared to the participants with younger age and shorter disease duration [73,78], worsening of motor and non-motor symptoms, such as rigidity, tremor, fatigue, pain, and concentration, has not been related to disease duration or severity [79]. In view of the above, it can be concluded that COVID-19 can cause different effects on PD via decreasing dopamine secretion, neurodegeneration, and can be a trigger for the developing or worsening of symptoms in PD and AD.

## 4. Conclusions

Since the COVID-19 pandemic, accumulating lines of evidence have revealed that besides common clinical manifestations, SARS-CoV-2 infection is associated with development or worsening of a variety of neuropsychiatric conditions. These can occur during or shortly after the onset of COVID-19. However, more recent longitudinal studies have revealed that COVID-19 increases the estimated risk of developing or worsening of neuropsychiatric conditions, such as mood, anxiety, psychotic disorders, dementia, insomnia, or even substance abuse disorders, after 6 months following COVID-19. These data indicate that close and long-term neuropsychiatric and cognitive monitoring of patients with COVID-19 are critical in order to diagnose these sequels and take appropriate therapeutic approaches as early as possible, and eventually improve patients’ and their families quality of life.

Although SARS-CoV-2 infection itself can be associated with the above-mentioned neuropsychiatric sequela, one should not overlook the multiple factors that contribute to the development or worsening of neuropsychiatric conditions during the COVID-19 pandemic. Overall, we can categorize these into three major domains: (i) the pandemic’s burden on the society acting as psycho-socio-economic stressors (e.g., deterioration of economic state, lockdown or quarantine, disruption of health care provision, and loss of job or family members due to COVID-19), (ii) disease-related reduction in quality of life (e.g., fatigue and breathlessness), and (iii) the impact of infection itself on brain function. These factors separately or in combination with each other may play critical roles in development or exacerbation of neuropsychiatric conditions during the pandemic. For instance, a population-based longitudinal study on 16,910 participants in the UK revealed an independent association between COVID-19 and increased risk of economic vulnerability among participants, measured by both household income sufficiency and sickness absence from work [80]. The economic recession resulting from the COVID-19 pandemic has adversely affected many people’s mental health and created new barriers for people already suffering from mental illness [81]. This is not only limited to COVID-19 pandemic, as such negative impacts on mental health and neuropsychiatric conditions have been seen in other global or regional shocks including wars [82,83,84], natural disasters [85,86], or other disease outbreaks, e.g., with Ebola [87,88], Zika virus [89], Middle East respiratory syndrome coronavirus (MERS) [90,91], and severe acute respiratory syndrome coronavirus (SARS-CoV) [90]. Social isolation due to lockdown or quarantine may have been particularly detrimental to individuals with neuropsychiatric and neurodevelopmental disorders, as social interaction and exercise may be used to regulate several symptoms of these disorders [46,92]. Data from an online questionnaire from 55,589 participants from 40 countries during the COVID-19 pandemic have revealed that lockdown significantly increases anxiety and depression at every degree of lockdown intensity, especially in combination with the presence of prior mental health issue [92]. This emphasizes the need for a proactive intervention to protect mental health of the general population but more specifically of vulnerable groups [92].

Almost over 2.5 years have passed since the COVID-19 pandemic; and as our knowledge about different neuropsychiatric manifestations directly or indirectly related to COVID-19 is improving, it overall highlights the critical need for global preparedness in the mental health sector during outbreaks of such infectious diseases. Important steps and innovations have been taken over the last 2 years to enable better service delivery to the affected populations. For instance, use of telemedicine and electronic prescriptions have become pivotal tools to be implemented globally [93]. This could be an essential element of continuity of care, especially during the lockdown or quarantine period. The promotion and empowerment of community-based mental health services especially in low-and-middle-income countries can also decrease the present treatment gap even during infectious disease outbreaks [94]. The development of global or local guidelines or consensus recommendations for such neuropsychiatric issues during or after the outbreak seems to be another effective way in reducing such psychiatric and mental health complications [94].

In the end, with an improvement of our knowledge about the pathophysiology of SARS-CoV-2 in brain dysfunction and neuropsychiatric conditions as well as the development of mediations targeting the infection itself or its related pathologic molecular signaling pathways, we may improve or even prevent the development of such neuropsychiatric manifestations. For instance, due to the presence of immune dysfunction and cytokine storm in COVID-19 patients, anti-inflammatory, immunomodulatory, or immunosuppressive medications have been tested or even in animal models of SARS-CoV-2 infections with variable benefits [95,96]. Vaccination has also created a new epoch in improving survival rate and acute critical illness related to COVID-19 [97]. Notably, a large retrospective cohort of 9479 individuals who developed COVID-19 despite SARS-CoV-2 vaccination, well-matched to controls, found that vaccination protected against severe acute illness, stroke, seizures, and psychotic disorders after breakthrough COVID-19 as assessed within 6 months post-vaccination. However, it may not protect from fatigue and other post-COVID-19 behavioral and cognitive symptoms [98]. Overall, research in this area is advancing. For instance, some studies have found an association between low neurotrophic factors and COVID-19-related neuropsychiatric complications [99]; thus, neurotrophic drugs such as cerebrolysin may serve as a potential therapeutic approach to improve neuropsychiatric manifestations of COVID-19 [100]. Clearly, more research studies and clinical trials are needed in this regard.

## Figures and Tables

**Table 1:** Incidence of anxiety and depressive psychiatric conditions in patients with COVID-19.

Population (Country)	Assessment	Psychiatric Conditions	Incidence	Ref.
126 (China)	Self-report questionnaire	Anxiety	22.2%	[11]
		PTSD	31%	
		Depression	38.1%	
402 (one month after hospital treatment) (Italy)	Self-report questionnaire	PTSD	28%	[12]
		Depression	31%	
		Anxiety	42%	
		Obsessive Compulsive Symptoms	20%	
		Insomnia	40%	
Prospective study in 44 hospitalized patients (USA)	HAD-A HAS-D	Depressive symptoms	29% (20% after 2 weeks)	[14]
		Anxiety	36% (9% after 2 weeks)	
		Acute stress disorder syndrome	25% mild-moderate (after two weeks)	
44,779 (the TriNetX Analytics Network)	Clinical diagnosis at 14–90 days (all first diagnoses)	Psychotic disorder	0.1%	[9]
		Any mood disorder	2%	
		Depressive episode	1.7%	
		Insomnia	1.9%	
		Dementia	1.6%	
236,379 (the TriNetX Analytics Network)	Clinical diagnosis at 6 months (all first diagnosis):	First dementia	0.67%	[8]
		Mood disorder	4.22%	
		Anxiety	7.11%	
		Psychotic features	0.42%	
		Insomnia	2.56%	
100 (UK)	Clinical evaluation	Any PTSD symptoms	41%	[15]
		Thoughts of self-harm	2%	
714 clinically stable patients (China)	Online PTSD questionnaire (PCL-C)	Significant post-traumatic stress symptoms	96.2%	[10]
57 (China)	Chinese version 9-item Patient Health Questionnaire (PHQ-9) and 7-item Generalized Anxiety Disorder (GAD-7) scale	Depression	29.2%	[13]
		Anxiety	20.8%	
153 (UK)	Clinical diagnosis	Affective disorder	2.6%	[16]
40,469 (the TriNetX Analytics Network)	Clinical diagnosis	Anxiety	4.6%	[6]
		Mood disorders	3.8%	
		Suicidal ideation	0.2%	
2150 hospital admitted patients (Spain)	Clinical diagnosis	Mood disorder	1.4%	[17]
		Anxiety-stress-adjustment disorder	11.9%	

## Data Availability

Not applicable.
